# Postharvest High-CO_2_ Treatments on the Quality of Soft Fruit Berries: An Integrated Transcriptomic, Proteomic, and Metabolomic Approach

**DOI:** 10.1021/acs.jafc.2c01305

**Published:** 2022-07-06

**Authors:** Irene Romero, M. Isabel Escribano, Carmen Merodio, M. Teresa Sanchez-Ballesta

**Affiliations:** Department of Characterization, Quality and Safety, Institute of Food Science, Technology and Nutrition (ICTAN), Spanish National Research Council (CSIC), Ciudad Universitaria, E-28040 Madrid, Spain

**Keywords:** fruit quality, gaseous treatments, omics technologies, postharvest, soft fruit berries

## Abstract

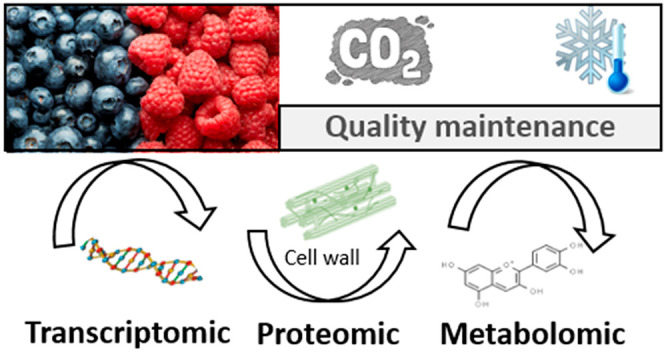

Soft fruits are appreciated for their taste qualities and for being a source of health-promoting compounds. However, their postharvest is affected by their high respiratory rates and susceptibility to fungal decay. Our aim here is to provide a perspective on the application of short-term high-CO_2_ treatments at a low temperature to maintain the postharvest quality of soft fruits. This work also suggests using a multi-omics approach to better understand the role of the cell wall and phenolic compounds in maintaining quality. Finally, the contribution of high-throughput transcriptomic technologies to understand the mechanisms modulated by the short-term gaseous treatments is also highlighted.

## Introduction

The development of new methods to reduce food loss and waste as part of the effort to improve food security and reduce global hunger has become one of the major challenges of this century. Furthermore, given the increasing concern about issues related to sustainable development and the environment, consumers are demanding that the use of chemical products is reduced significantly. Therefore, incorporating non-contaminant postharvest technologies to reduce the use of agrochemical compounds is vital to maintain the quality and avoid the loss of fruits and vegetables during postharvest storage.

Fresh soft fruit berries, such as blueberries (*Vaccinium* spp.) and raspberries (*Rubus idaeus* L.), are gaining popularity because of their nutritional and health benefits, and their economic value is increasing accordingly. Despite the coronavirus pandemic, declining revenues, and complicated logistics, trade in soft fruit berries flourished in 2020. In this regard, blueberries were the most demanded, being the berry whose trade is growing most rapidly (around 16–18% in 2020 and exceeded $ 4.5 billion). This means that the volume of world trade in fresh blueberries is already at least $1.2 billion higher than the volume of fresh strawberries.^1^

The shelf life of soft fruit berries at room temperature varies considerably among cultivars, but in general, it is limited as a result of their high respiration rates, fragile structures, and high susceptibility to fungal decay.^2^ To overcome these disadvantages, it is recommended to store fruits at a temperature near 0 °C to extend their postharvest storage. However, cold storage triggers total decay and softening and, in the case of blueberry, pedicle pitting as well as pericarp and pulp adhesion.^3^ Moreover, the absence of a protective skin in raspberries and strawberries favors the induction of water and weight losses, reducing their postharvest life even at a low temperature. As a result, low-temperature storage under air conditions is not able to extend the storage life of soft fruit berries more than 20 days, with this period lasting only about 7 days in the case of raspberries.^4^ Therefore, for longer storage periods, combining storage at low temperatures with other technologies is necessary. Physical treatments that modify the storage atmosphere, commonly lowing O_2_ or/and increasing high CO_2_, in combination with low-temperature storage, such as controlled atmosphere (CA) and modified atmosphere packaging (MAP), have been used to extend the postharvest life of blueberries, raspberries, and strawberries.^4^ However, prolonged exposure to very low O_2_ concentrations and very high CO_2_ levels can result in off-flavors and off-odors as a result of anaerobic respiration and fermentative metabolism.^5^ Hence, applying gaseous treatments at low temperatures for short periods (1–3 days) could create optimal conditions for maintaining berry quality during postharvest and avoiding the adverse effects mentioned above. Moreover, an important aspect to consider is that the application of these short-term gaseous treatments could reduce costs compared to traditional CA or MAP systems because the time of exposure to non-atmospheric conditions is reduced. If a literature review were undertaken with the aim of learning about the effect of short-term gaseous treatments on soft fruit, it would be seen that most of the research has been performed on strawberries,^6,7^ and thus, blueberries and raspberries are an area worth exploring. Consequently, the aim of this study is to offer a new perspective on blueberry and raspberry postharvest through the application of short-term gaseous treatments at a low temperature, with a particular focus on the aspects that need to be considered to carry out a full evaluation of their efficacy.

## Short-Term High-CO_2_ Treatments To Maintain Blueberry and Raspberry Fruit Quality during Postharvest

It is known that CO_2_ gas concentrations ranging between 15 and 20% reduce fungal attack, respiration, water loss, and softening of fruit berries, thereby extending their postharvest life.^2,8^ In reference to short-term treatments, currently, only one recently published study has proven that a short-term treatment with 15% CO_2_ for 3 days, followed by air storage at 1 °C for 11 days, was effective in maintaining color parameters and firmness in raspberries.^9^ The first aspect to consider is the duration of the treatment and the concentration of CO_2_ used because, although these are short treatments, these two parameters (time and dose) are known to affect the response of the fruit. For example, off-flavors develop when CO_2_ concentrations exceed the tolerance threshold during a 3 day short-term gas treatment to maintain strawberry quality. Thus, while the application of a 3 day treatment with 20% CO_2_ is effective in maintaining strawberry quality, higher concentrations (40%) administered for the same time lead to the accumulation of fermentative products generating off-flavor.^10^ In the case of blueberries and raspberries, as a first step, both the time of exposure and CO_2_ dose conditions should be optimized to improve their storability. Moreover, to obtain a real understanding of the effect of short-term gaseous treatments, it would be necessary to clarify whether they are cultivar-dependent or not. Although it is well-known that short-term treatments are effective in maintaining the postharvest quality of different cultivars of grape berries, the molecular responses and metabolites elicited were cultivar-specific.^8^

Another important aspect to consider when designing short-term gaseous treatments for raspberries and blueberries is that the signals that trigger their ripening have, up until now, been poorly understood, and in fact, there is some controversy as to whether these fruits should be classified as climacteric or non0climacteric. Although raspberries have been classified as non-climacteric, the pattern of ethylene production in these fruit shows a constant increase during ripening, quite different from other non-climacteric fruits, such as strawberries and grapes.^11^ On the other hand, even though blueberries have been classified as climacteric fruit, there are certain discrepancies in the literature. Unlike many climacteric fruits, which can be harvested at a pre-climacteric stage of development and ripened closer to the market in a controlled manner, blueberries depend upon the plant for assimilates and should be harvested as near to commercial maturity as possible because their organoleptic attributes do not improve after harvesting, particularly sweetness, because they lack starch reserves. Thus, all of these variables must be taken into account to perform short-term gaseous treatments in raspberries and blueberries.

## Elucidation of the Mechanisms through a Multi-omic Approach

### Importance of the Cell Wall: A Proteomic Approach

Fruit softening and postharvest decay are two main problems causing deterioration in the quality and appearance of soft fruit and contribute significantly to their loss. It is believed that disassembly of cell wall polymers and the dissolution of the middle lamella, as a result of the coordinated expression of several gene families encoding cell-wall-modifying enzymes,^12^ are mainly responsible for softening and could contribute to pathogen attack by reducing the strength of the cell wall, the main barrier against tissue colonization. Thus, it is imperative to investigate how the cell wall structure and composition relate to properties of blueberries and raspberries and how these are modified in response to the postharvest conditions in these fruits. Different studies have addressed the firmness trait of blueberries, focusing on physiological and molecular changes at the onset of ripening as well as individual differences.^13^ However, to improve soft fruit postharvest quality, we need to further explore cell-wall-modifying genes and proteins in response to postharvest treatments to be able to identify all molecular markers linked to maintaining firmness through the use of short-term gaseous treatments.

Different cell wall models have emphasized the relevance of non-covalent interactions between polymers, which may respond dynamically to developmental and environmental conditions.^14^ In this context, it has been shown that structural *O*-glycoproteins, extensins, and arabinogalactan proteins form complexes with pectin and xylans by ionic interaction and covalent cross-links.^15^ Expansins induce non-enzymatic cell wall loosening by disrupting non-covalent interactions between cellulose microfibrils and the hemicellulose polymers that coat them in the wall.^16^ In this sense, the macromolecular structure–function relationships of cell wall polymer–protein networks as well as the mechanisms for their assembly and subsequent remodeling remain an active area of research. Current and emerging immunocytochemical and immunohistochemical techniques, using novel and well-designed specific probes, are being employed for monitoring the dynamics and precise localization of cell wall structural polysaccharides and *in situ* proteins within complex tissues. It is well-known that strawberries exposed to 20% CO_2_ are firmer than those stored in air at a low temperature because cell-to-cell adhesion and the integrity of the middle lamella are maintained as a result of the treatment with high CO_2_ levels^17^ and a downregulation of pectin methylesterase proteins occurs.^18^ Additionally, the presence of major enzymes and genes involved in the degradation of pectin, the main component of middle lamella during blueberry postharvest, has been reported.^19^ However, the effect of high levels of CO_2_ on structural proteins has not yet been characterized, and there is little information about the spatial and temporal localization of these structural cell wall proteins. Hence, their precise localization within the cell wall would require further investigations at the cytological and histological levels. Furthermore, the role of expansins in blueberries and raspberries during ripening has been studied,^20,21^ but how they are distributed spatially in soft fruit berries is relatively unknown, a factor that could help better explain the function of this family of proteins. Thus, further studies on polymer–protein assembly are required to advance our knowledge of the effect of high levels of CO_2_ at low temperatures on the maintenance of soft berry textures, which is a research area that also needs to be deepened.

The cuticle plays a significant role in the softening process, in reducing water loss, and in protecting fruits from pathogen invasion. Postharvest changes in the fruit cuticle have not received much interest until recently, and while not abundant, the majority of the published reports indicate that fruit cuticles continue to evolve after harvest and that a common pattern of change cannot be expected for different species or even cultivars.^22^ Softening of Duke and Brigitta blueberry fruits stored at 0 °C has been shown to be highly correlated to cuticle composition, indicating its relevance on this attribute.^23^ Also, despite the important implications for postharvest fruit quality, little effort has been devoted to the study of cuticle formation, especially from a biochemical and molecular perspective. Therefore, in our view, it is essential to investigate whether high CO_2_ levels alter cuticle composition during storage at a low temperature and whether these changes are related to the reduced water loss associated with short-term high-CO_2_ treatments.

### Importance of Phenolic Compounds: Transcriptomic and Metabolomic Approaches

Another essential quality factor to be maintained in soft berries during postharvest is their functional properties. In fact, these fruits are especially appreciated because of their high content in antioxidants. The most common antioxidants in soft fruit berries are vitamin C and polyphenols, such as phenolic acids, flavonoids (anthocyanins, flavanols, and flavonols), and tannins. These compounds can be easily altered by many factors, including certain postharvest storage conditions. Despite the amount of studies on the regulation of phenylpropanoid metabolism, specifically anthocyanins and other flavonoids in many plant species, little is known in soft berries. With regard to these fruits, most studies have been performed during their stages of development and ripening, but information related to the effect of postharvest conditions is very limited. At this point, it is important to note that genes required for the biosynthesis of flavonoids are controlled predominantly at the transcriptional level. Different protein superfamily members mediate the transcriptional regulation of the flavonoid biosynthetic pathway, such as the MYB and basic helix–loop–helix (bHLH) transcription factors and the conserved WD40 repeat (WDR) proteins.^24^ However, nothing is known about the effect of short-term gaseous treatments on transcription factors and proteins mediating the transcriptional regulation of flavonoids. Thus, new perspectives on soft berries postharvest should include not only the study of flavonoid accumulation but also their transcriptional regulation.

Different works have analyzed the content of polyphenols as well as the antioxidant capacity of soft berries cultivated in different locations and during ripening. Results obtained by various researchers on the impact of different postharvest treatments on the antioxidant capacity of polyphenols in soft fruit berries were contradictory.^25,26^ In this sense, it should be noted that most studies on the total antioxidant capacity of fruit samples have been carried out by individually determining the antioxidant compounds or by monitoring the reaction between them and certain test reagents. These methods measure a method-characteristic capacity but do not provide direct mechanistic information.^27^ Lately, electrochemistry has begun to play a significant role in the study of the antioxidant capacity of fruit and the characterization of fruit cultivars according to the postharvest storage conditions, both of which require further investigation. In recent years, an electrochemical methodology using solid-state voltammetry has been developed. This methodology has also permitted the reactivity of phenolic compounds with reactive oxygen species to be monitored after their *in situ* electrochemical generation.^28^ Moreover, it allows table grape samples to be differentiated depending upon the storage atmosphere (air or short-term gaseous treatments).^29^ The fact that this voltammetric method exploits the electroactive character of polyphenolic compounds abundant in raspberries and blueberries opens a new perspective in studying how the application of postharvest treatments might affect their antioxidant capacity.

### Contribution of High-Throughput Transcriptomic Technologies to Soft Fruit Postharvest

**Figure 1:**
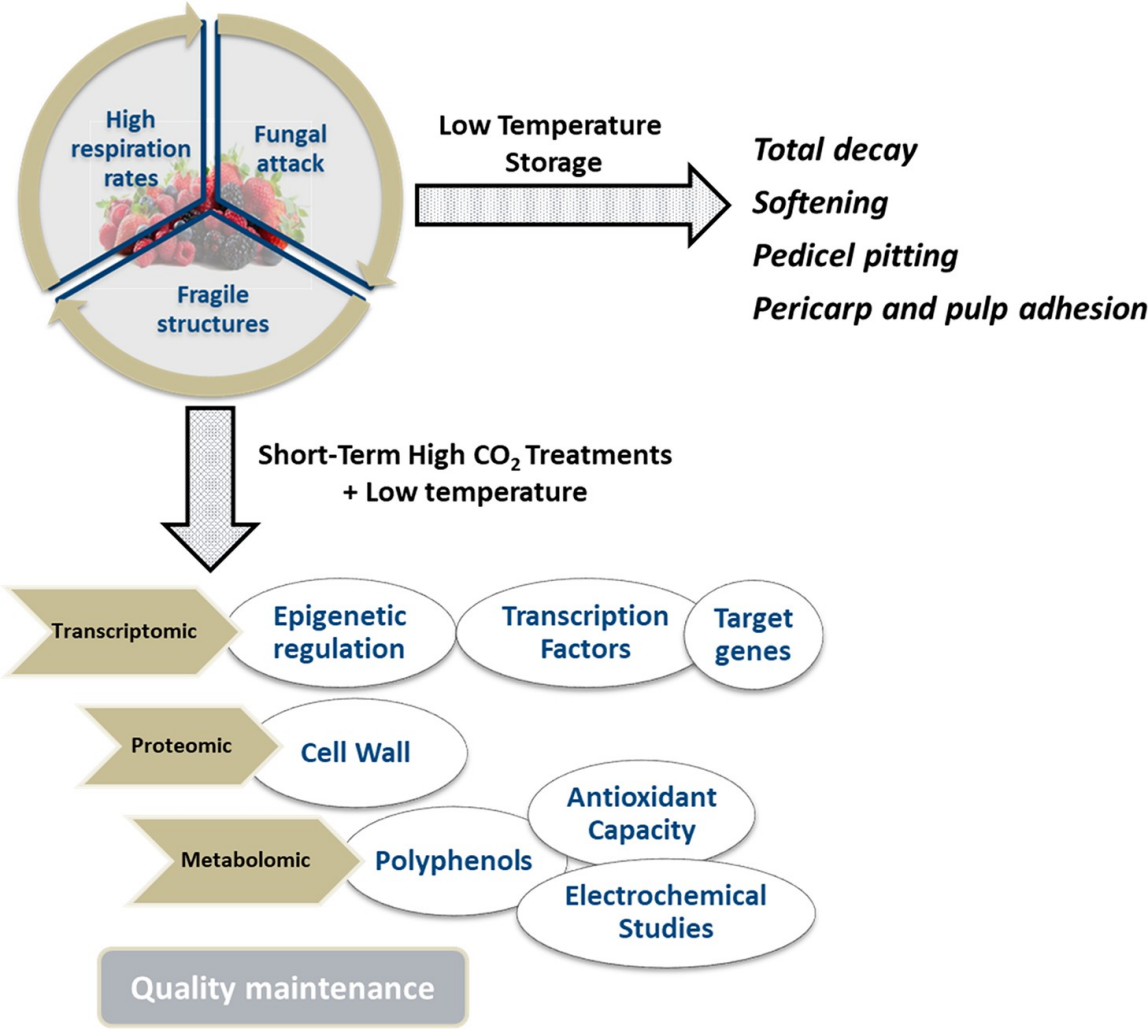
Schematic of future prospects for a multi-omics approach aiming to determine the effect of short-term CO_2_ treatments in soft fruit berries.

High-throughput omics techniques using transcriptomic approaches have become a viable option to support traditional postharvest research. Recent advances in sequencing and computational technologies have greatly facilitated the generation of a large amount of sequence data in a relatively fast and cheap manner. Moreover, transcriptome sequencing (RNA-seq) has opened many doors for high-throughput discovery of genes and genetic markers and quantifying gene expression in non-model species, such as *Rubus* and *Vaccinium* genus, which lack reference genome information. Most RNA-seq studies concerning soft fruit berries have been performed to characterize developmental and ripening processes. With regard to postharvest, recently, a *de novo* RNA-seq analysis was performed on blueberries with the purpose of characterizing the response of these fruits to cold storage.^30^ The study highlighted the relevance of genes involved in cell wall metabolism, synthesis of wax compounds, and biotic and abiotic stress. However, no studies on the effect of gaseous treatments have been conducted until now. Consequently, comparative RNA-seq analyses could be useful as a means to undertake an in-depth study of the molecular mechanisms modulated by low-temperature gas treatments used to maintain the quality of soft fruit berries during postharvest. (Figure 1). Currently, attention is being paid to the role of epigenetic regulation of gene expression as a modulating mechanism of genome activity and its impact on fruit quality. To date, almost all studies on epigenetics in the postharvest field have focused on the modulation of pathways involved in fruit development, including ripening and senescence, but to our knowledge, no studies have been conducted on blueberries and raspberries. A new perspective would involve a holistic analysis of epigenetic modification sites that could be relevant to gene expression profiles and fruit quality during postharvest. Therefore, to translate all of this information into a potential application for soft fruit postharvest, future research in this field should aim to develop a more comprehensive experimental design that considers multiple regulatory levels, including transcriptional networks, post-transcriptional regulation, and epigenetics. This will require data mining, high-throughput approaches, and the development of user-friendly tools to implement multi-omics strategies.
